# In Situ Observation of Deformation in a Sn-3Ag-0.5Cu/Cu Solder Joint Using High-Voltage Transmission Electron Microscopy

**DOI:** 10.3390/ma18163925

**Published:** 2025-08-21

**Authors:** Kazuhiro Nogita, Xin Fu Tan, Jiye Zhou, Stuart D. McDonald, Keith Sweatman, Flora Somidin, Guang Zeng, Hiroshi Maeno, Kazuhiro Yasuda, Christopher M. Gourlay

**Affiliations:** 1 Nihon Superior Centre for the Manufacture of Electronic Materials (NS CMEM), School of Mechanical and Mining Engineering, The University of Queensland, St. Lucia, QLD 4072, Australia; xin.tan@uq.edu.au (X.F.T.); jiye.zhou@uq.edu.au (J.Z.); s.mcdonald1@uq.edu.au (S.D.M.); k.sweatman@nihonsuperior.co.jp (K.S.); 2 Nihon Superior Co., Ltd., Osaka 564-0063, Japan; 3 Electronic Packaging & Thin Film Materials Research Group, Centre of Excellence Geopolymer and Green Technology (CEGeoGTech), Universiti Malaysia Perlis (UniMAP), Taman Muhibbah, Jejawi, 02600 Arau, Perlis, Malaysia; flora@unimap.edu.my; 4 School of Materials Science and Engineering, Central South University, Changsha 410083, China; g.zeng@csu.edu.cn; 5 The Ultramicroscopy Research Center, Kyushu University, Fukuoka 819-0395, Japan; maeno.hiroshi.551@m.kyushu-u.ac.jp (H.M.); yasuda.kazuhiro.967@m.kyushu-u.ac.jp (K.Y.); 6 Department of Applied Quantum Physics and Nuclear Engineering, Kyushu University, Fukuoka 819-0395, Japan; 7 Department of Materials, Imperial College London, London SW7 2AZ, UK; c.gourlay@imperial.ac.uk

**Keywords:** electronics packaging, Pb-free solder, in situ deformation, high-voltage transmission electron microscopy

## Abstract

For reliable electronics, it is important to have an understanding of solder joint failure mechanisms. However, because of difficulties in real-time atomistic scale analysis during deformation, we still do not fully understand these mechanisms. Here, we report on the development of an innovative in situ method of observing the response of the microstructure to tensile strain at room temperature using high-voltage transmission electron microscopy (HV-TEM). This technique was used to observe events including dislocation formation and movement, grain boundary formation and separation, and crack initiation and propagation in a Sn-3 wt.%Ag-0.5 wt.%Cu (SAC305) alloy joint formed between copper substrates.

## 1. Introduction

Tin (Sn)-based alloys that wet and react with copper (Cu) substrates to form solder joints remain an essential part of electronics manufacturing. During the process of soldering to Cu substrates, a multi-phase structure is formed that typically includes intermetallic compounds (IMCs) such as Cu_6_Sn_5_ and Cu_3_Sn. In service these joints are subjected to mechanical stresses that result from mismatches in the coefficients of thermal expansion (CTE) of the components and the substrates to which they are attached during the thermal cycles generated by the operation of the device and/or the environment to which the device is exposed [1,2,3,4,5,6,7,8,9,10]. The solder joint failure mechanism is complex and dependent on the stress, the rate at which it is applied, the temperature, and the previous thermal history, as well as the composition of the alloy and substrate [9,10]. Failure may occur between the substrate and the IMC layer, between the solder and the IMC layer, or in the bulk solder [11,12,13,14,15,16]. Numerous researchers have found a relationship between the recrystallisation of the β-Sn phase that occurs during thermal cycling and the path followed by the cracks that ultimately cause joint failure [10,17]. Because of the high homologous temperature, recovery and recrystallisation in areas of high damage accumulation can occur even at room temperature, so the observations made in the testing reported here are relevant to the performance of solder joints in working electronics [18,19,20].

The current model for solder joint failure [10,17] has the following stages: (1) damage accumulation in areas of the solder joint subject to maximum shear strain; (2) recrystallisation of the tin in the areas of accumulation damage; (3) grain boundary sliding and grain rotation in the recrystallised area; and (4) crack initiation and propagation at the grain boundaries. While this work has drawn attention to the role that strain-induced recrystallisation can play in solder joint failure mechanisms, it is based on examination of cross sections of joints taken from the thermal cycling chambers at intervals during the expected time to failure. While representing a significant advance in our understanding of the solder joint failure mechanism, these studies can tell only part of the story of solder joint failure in service. Despite advances in atomic scale in situ analysis [21,22,23], the mechanisms of recrystallisation and crack propagation during solder joint deformation are not fully understood. As well as contributing to solder joint failure, it has been reported that recrystallisation can play a role in the processes that result in the growth of tin whiskers [24,25]. Tin whiskers are a recognised cause of failure in circuitry and a better understanding of recrystallisation could therefore be a useful contributor to the formulation of whisker-resistant solder alloys. An understanding of the recrystallisation mechanisms and the relationship to crack initiation and propagation in solder joints based on real time in situ observation could provide a stronger basis for the formulation of solder alloys that can deliver more reliable interconnects in electronic circuitry.

This paper reports the development of a method of in situ observation of the processes that occur within the microstructure of a model solder joint that lead to its eventual failure. That the observations are consistent with the failure model that has been developed on the basis of studies of the cross sections of solder joints subjected to thermal cycling means that the model can be used with confidence as the basis for the development of higher-reliability solder joints that are needed to support the electronics on which the world increasingly relies.

## 2. Materials and Methods

### 2.1. Sample Preparation

**Figure 1:**
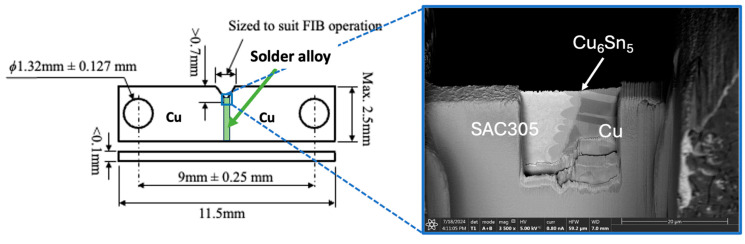
Sample geometry for in situ tensile loading experiment in high-voltage TEM. An electron-transparent region is prepared at the tip of the notch using FIB.

Two laser-cut notched Cu plates (thickness of <0.1 mm) were hand-soldered using Sn-3 wt.%Ag-0.5 wt.%Cu (SAC305) solder (supplied by Nihon Superior Co. Ltd., Osaka, Japan) with a soldering iron set at 350 °C to fabricate a Cu/SAC305/Cu solder joint, as shown in Figure 1. An electron-transparent region, (approximately 0.5 μm in thickness) was prepared at the tip of the notch using a focused ion beam (FIB) milling process (FEI, Scios FIB—Dual Beam SEM). A platinum-rich precursor was deposited to form a protective layer.

### 2.2. In Situ Tensile Testing Conditions

**Figure 2:**
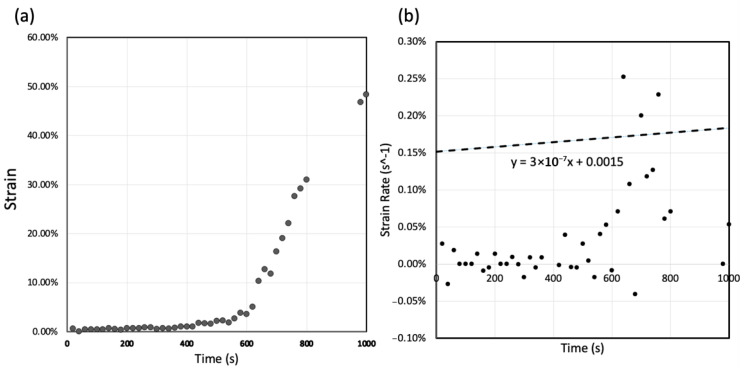
(**a**) Strain (%) and (**b**) average strain rate (%s^−1^), measured from Supplemental Video S1.

The sample was prepared for TEM observation by placing it on a holder (Single Tilt Heating Straining Holder, Model 672, Gatan, Pleasanton, CA, USA) in an HV-TEM (JEM-1300NEF, JEOL, Tokyo, Japan) operated at an accelerating voltage of 1250 kV with the Omega-type energy filter. Incident electron beam dose measurements were performed with an in-beam Faraday cup inserted between the specimen and the fluorescent screen in a region without the specimen. A flux of 2.42 × 10^−10^ A with an electron beam diameter of 21.3 μm was used for the low-magnification (×2000) observation. Tensile strain was applied at a crosshead speed of 1.0 μm/s. Due to the complex sample geometry, the loading is not purely tensile, but the results nevertheless provide a new insight into the failure mechanism in solder joints. A video was recorded at a rate of 10 frames per second during tensile loading. The average strain rate of the sample was measured in the direction of crosshead travel from the recorded video (Supplemental Video S1) as shown in Figure 2 and was approximately 0.0015 s^−1^.

## 3. Results

**Figure 3:**
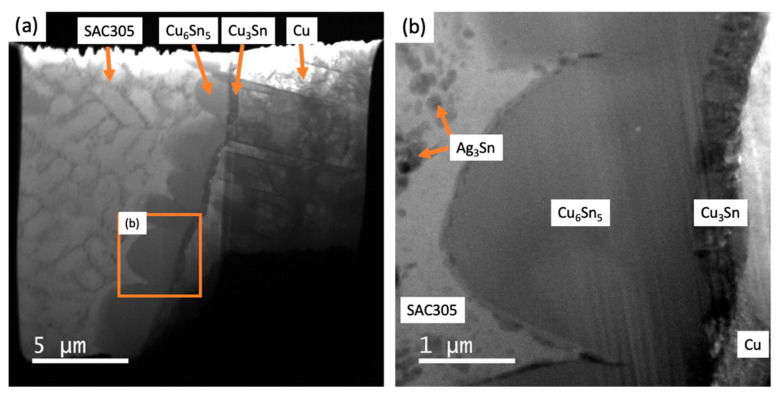
Energy-filtered TEM images of the solder joint observation area before tensile loading, at (**a**) low magnification and (**b**) high magnification. Tensile loading is in the horizontal direction at a crosshead speed of 1.0 μm/s.

Energy-filtered TEM images of the observation area in the SAC305/Cu_6_Sn_5_/Cu_3_Sn/Cu sample before tensile loading show the dendritic primary Sn within the SAC305 solder alloy microstructure (in Figure 3a) along with the layer of Cu_6_Sn_5_. At higher magnification (in Figure 3b), the eutectic Ag_3_Sn phase is visible in the interdendritic regions, along with a 2–3 μm thick, scalloped layer of Cu_6_Sn_5_ and a sub-micron-thick Cu_3_Sn layer adjacent to the Cu substrate. This distribution of phases is typical of the joint microstructure that results from the reaction between liquid SAC305 and the Cu substrate followed by solidification during soldering [26].

**Figure 4:**
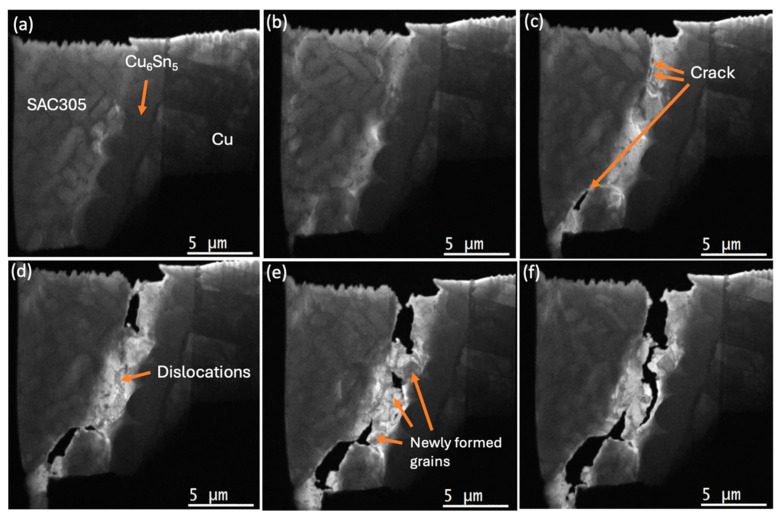
Still images from Supplemental Video S2a. In situ TEM observations of the solder joint with a tensile loading at a crosshead speed of 1.0 μm/s. (**a**,**b**) Sample thickness (indicated by brightness) is reduced in the area of the SAC305 solder adjacent to the Cu_6_Sn_5_; (**c**) crack initiation occurs in several regions; (**d**) dislocation accumulation occurs at the crack propagation front; and (**e**,**f**) crack propagation occurs in the newly formed grain boundaries of the Sn phase. The tensile loading direction is horizontal in the figures.

**Figure 5:**
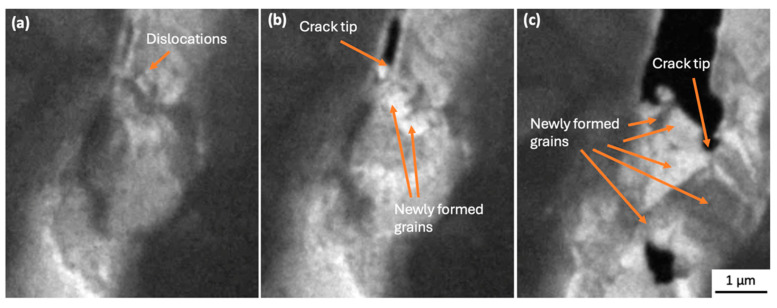
Still images from Supplemental Video S2a. Crack propagation process, (**a**) dislocation accumulation, (**b**) crack formation and nucleation of new grains, and (**c**) crack propagations along new grain boundaries. The tensile loading direction is horizontal in the figures.

The entire experimental in situ observations are provided as Supplemental Video S1 (×30). Figure 4a–f and Figure 5a–c, and Supplemental Video S2a (×4), show the in situ TEM during tensile loading of the SAC305/Cu_6_Sn_5_/Cu solder connection, revealing deformation, dislocation accumulation, crack formation and propagation, and the related formation of recrystallised grain boundaries. The observations were successful in identifying the moment of crack initiation in the SAC305 solder alloy, which occurred in the β-Sn adjacent to the Cu_6_Sn_5_ intermetallic, but not within Cu_6_Sn_5_ or Cu_3_Sn or the interface between the SAC305 solder and Cu_6_Sn_5_. This mode of failure is similar to that observed in SAC305 solder joints to ball grid array (BGA) packages during thermal cycling [6,7,8,9,10,15].

**Figure 6:**
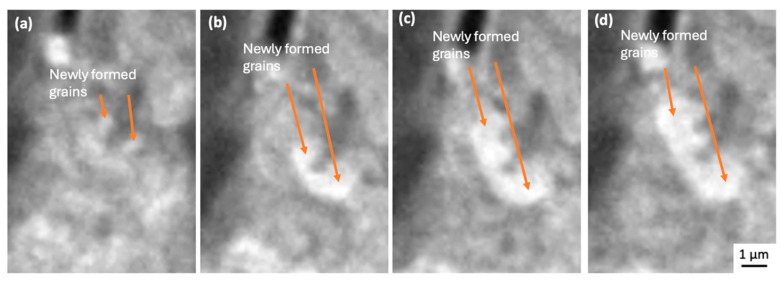
Still images from Supplemental Video S2b. Recrystallised grains nucleate at dislocation pile-up at crack tip; (**a**) nucleation of new grains; (**b**–**d**) growth of new grains. The tensile loading direction is horizontal in the figures.

The crack path in the area of dislocation accumulation adjacent to the Cu_6_Sn_5_ intermetallics, followed the newly formed grain boundaries, which were revealed by variations in contrast in the TEM images with the strain due to the dislocations and new grain boundary movement, as well as bend contour movement, displayed dynamic behaviour during tensile loading. Due to the time resolution limit of 10 frames per second, it was difficult to record the moment of nucleation of new grains (see Figure 6); however, crack initiation and propagation along with dislocation accumulation were clearly observable (e.g., Figure 4c–f).

**Figure 7:**
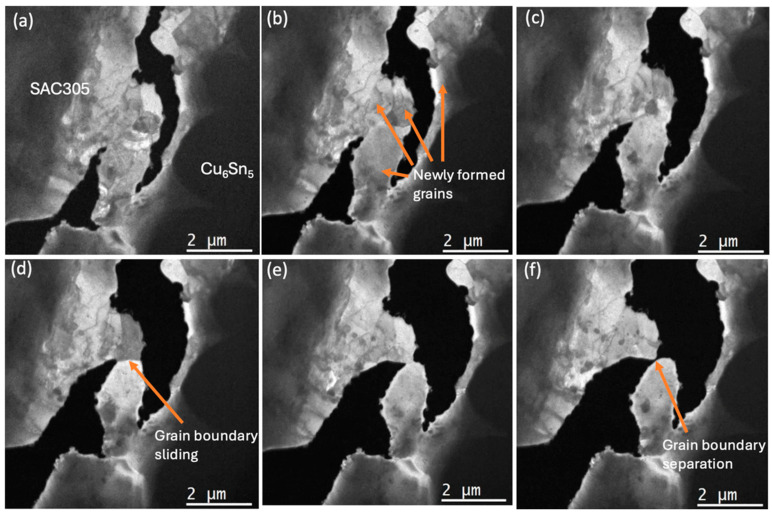
Still images from Supplemental Video S2b. In situ energy-filtered TEM images of the solder joint with tensile loading at a crosshead speed of 1.0 μm/s, after crack formation. The process of crack propagation along with sliding at the boundaries of recrystallised grains is apparent. The strain progresses from (**a**–**e**) until complete separation at (**f**) and the tensile loading direction is horizontal in the figures.

**Figure 8:**
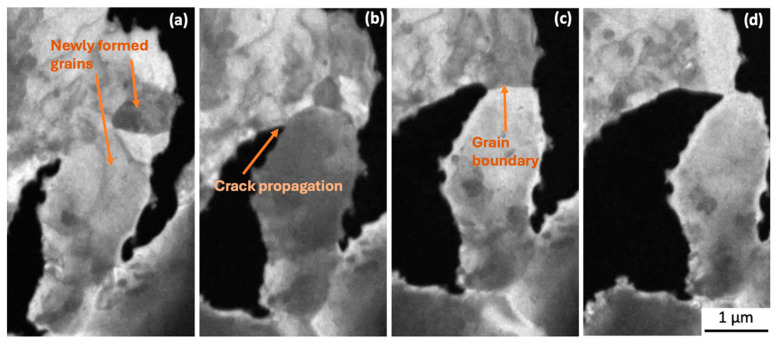
Still images from Supplemental Video S2b. Higher magnification of Figure 4. Crack propagation at newly formed grain boundary (**a**,**b**) followed by sliding prior to separation (**c**,**d**). Tensile loading direction is horizontal in the figures.

**Figure 9:**
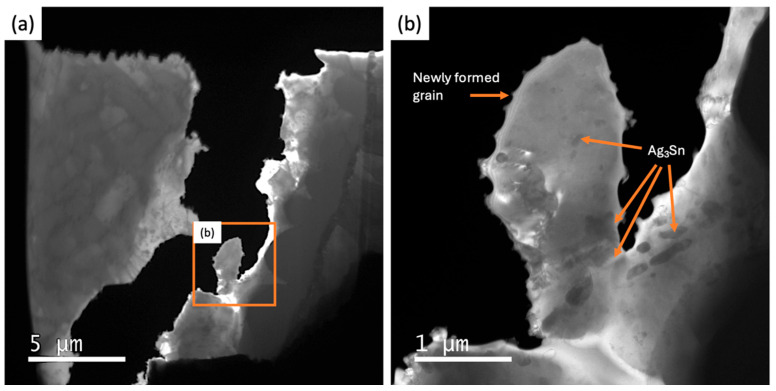
Energy-filtered TEM images of newly formed grains after total separation during testing. (**a**) Low magnification and (**b**) higher magnification of square marked region of (**a**). Newly formed grains containing some Ag_3_Sn particles.

Figure 7a–f, Figure 8a–d and Figure 9a,b and Supplemental Video S2b (×4) show higher-magnification imaging of what appear to be new grains that are growing in areas of dislocation accumulation. It can be seen that the newly formed grain boundary sliding and subsequent grain separation together represent the crack propagation event. Bieler et al. [5] reported that cracks form at random recrystallised boundaries, which then spread through recrystallised regions. Because of the difficulty in determining the orientation of the newly formed grains, we cannot confirm that these grains are a result of recrystallisation that occurred during testing. However, the observations of crack initiation and propagation are consistent with the recrystallisation model.

## 4. Discussions

### 4.1. Electron Beam Interactions with Sample

#### 4.1.1. Electronic Stopping Power and Beam Heating

The electronic stopping power in Sn (density: 7.31 g/cm^3^), which is related to the electron beam heating, has been calculated using ESTAR (Stopping Power and Range Tables for Electrons) from the NIST Standard Reference Database 124 “Stopping-Power & Range Tables for Electrons, Protons, and Helium Ions” [27].

**Figure 10:**
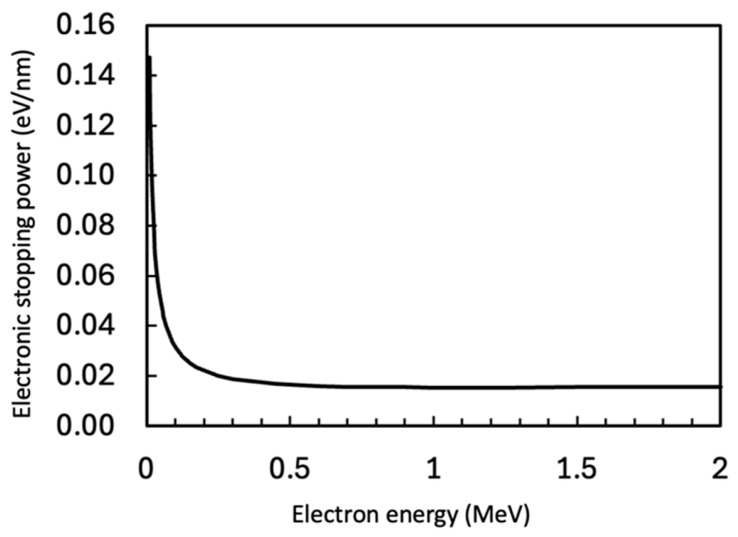
Electronic stopping power in Sn calculated using ESTAR (Stopping Power and Range Tables for Electrons) from NIST Standard Reference Database 124 “Stopping-Power & Range Tables for Electrons, Protons, and Helium Ions” [27].

Figure 10 shows the relationship between incident electron beam energy (acceleration voltage of TEM) (MeV) and the electronic stopping power (eV/nm). When the beam energy is 200 keV (conventional TEM), the electronic stopping power is 0.02 eV/nm, while at 1250 keV, it is calculated as 0.015 eV/nm. The results indicate 30% less beam heating effect for the current experiments with 1250 keV condition compared with a conventional 200 keV TEM condition. Also, Sn is metallic, with relatively high thermal conductivity (66.8 W/m·K); therefore, the effect of beam heating on the sample is estimated to be negligible.

#### 4.1.2. Knock-On Damage

**Figure 11:**
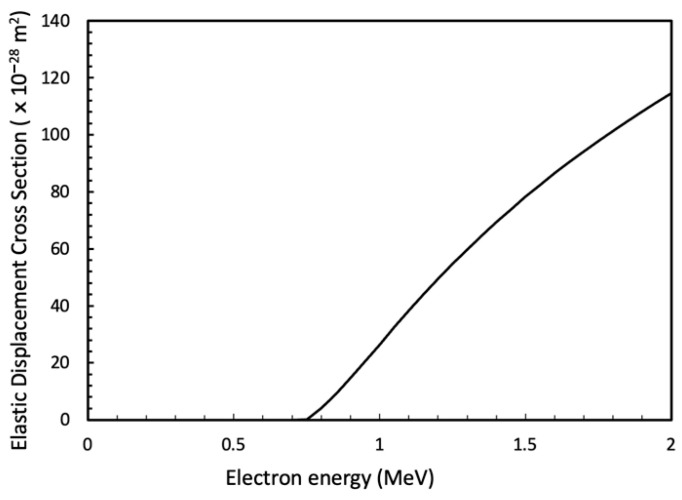
Displacement cross section for Sn metal as a function of electron energy with a displacement energy of 24 eV [30] using a SMOT/POLY code [28,29].

The incident electron beam was sufficiently energetic to displace Sn atoms from their lattice sites during observation. Therefore, we calculated the elastic displacement damage rate (dpa/s) using the SMOTT/POLY computer code, based on Mott cross sections [28,29]. Figure 11 shows the elastic displacement cross section for Sn as a function of incident electron beam energy. During observation at ×2000 magnification (electron beam flux: 2.42 × 10^−10^ A, beam diameter: 21.3 μm), and with a threshold displacement energy for Sn of 24 eV [30], the radiation damage caused by the incident beam was identified to be 6.76 × 10^−6^ dpa/s. No dislocation loops or other defect types, such as dot contrasts, were observed during the measurements. Therefore, we believe that the effects of radiation damage on dislocation accumulation during tensile loading and crack formation were minimal.

### 4.2. Dynamic and Static Recrystallisation

In general, recrystallisation temperatures are between 0.4 and 0.7 T_m_ (where T_m_ is the melting temperature in Kelvin). For Sn, which has a melting point at 231.9 °C or 505.05 K, 30 °C or 303.15 K corresponds to a homologous temperature of 0.6 Tm, which means that dynamic recrystallisation will spontaneously occur around room temperature if tensile loading results in sufficient strain in the sample [19,20].

During continuous tensile loading, multiple dislocations could be seen in front of the tip of the advancing crack and then migrating to nearby subgrain boundaries, growing newly formed grains as well as nucleating new subgrains (Figure 6). The limitations of the sample geometry and condition meant that electron diffraction or electron backscatter diffraction (EBSD) could not be used to confirm that grain boundaries were of a high angle. Nevertheless, it is clear that whatever their origin, the newly formed grains, sliding along with newly formed grain boundaries, are playing a part in crack initiation and propagation (Figure 7a–f, Figure 8a–d and Figure 9a,b and Supplemental Video S2b (×4)). This behaviour is consistent with the process of discontinuous recrystallisation (DDRX) (in Figure 6), similar to refs. [5,31,32,33].

There are two main mechanisms in the process of recrystallisation [31,32,34]: (i) discontinuous or classical dynamic recrystallisation (DDRX), occurring by the nucleation and growth of new grains rapidly consuming the surrounding strain-hardened matrix, and (ii) continuous dynamic recrystallisation (CDRX), involving the generation of new grain boundaries by the progressive misorientation of neighbouring subgrains by dislocation climbing. Since we found new grains at the tip of the crack and a low density of dislocations after new grain formation, the mechanism operating is expected to be (i) a DDRX mechanism, as evident in Figure 4d,e, Figure 5 and Figure 6, and Supplemental Video S2a. This mechanism corresponds well with the report by B. Zhou et al. [33] that shows the recrystallisation process at a solder/substrate interface during thermal cycling. As shown in Figure 2, the strain rate increased around the time of initial crack formation at approximately 600 s after tensile loading. How variations in strain rate may affect recrystallisation dynamics or crack initiation mechanisms is difficult to establish with the current experimental set up. The development of this method is continuing, with the objective of finding answers to those questions.

There are some Ag_3_Sn particles in the new grains, but most of these particles are present along the grain boundaries or in areas that have not undergone recrystallisation, as can be seen in Figure 9. This result is consistent with previous work on thermally cycled BGAs that reported that coarsened Ag_3_Sn particles are often located at recrystallised grain boundaries, whereas the interior of recrystallised grains are, in many cases, particle-free zones [10]. Ag_3_Sn is well recognised as a “particle strengthening” compound that acts as an obstacle to dislocation movement. Slowing dislocation movement slows the damage accumulation that drives recrystallisation [18]. This suggests that the design of crack-resistant BGAs should further consider the effect of IMC particles on both recrystallisation and crack growth processes, including interactions between recrystallisation and accelerated particle coarsening. Further studies comparing solder alloys with and without particle strengthening by Ag_3_Sn will be required to determine the effect that these particles have on recrystallisation.

## 5. Conclusions

In conclusion, we successfully fabricated a mechanical test specimen with a microstructure representative of a typical SAC305 solder joint that was locally thinned for in situ TEM observation using HV-TEM during tensile loading at room temperature. The phenomena observed were dislocation accumulation, crack initiation, and propagation along grain boundaries. Also observed were what appeared to be new grains that formed in areas of high dislocation concentration, but in the work reported here it was not possible to carry out the crystallographic experiments that would confirm whether they are new grains. In the future this technique will be used to obtain evidence of recrystallisation occurring during both static and dynamic loading over a range of temperatures and strain rates in a variety of solder alloys.

## Data Availability

The original contributions presented in this study are included in the article/Supplementary Material. Further inquiries can be directed to the corresponding author.

## Supplementary Materials

The following are available online at https://www.mdpi.com/article/10.3390/ma18163925/s1: Video S1. In situ TEM video for whole tensile loading process; Video S2a. In situ TEM video for dislocation accumulation at crack tip and nucleation of new grains; Video S2b. In situ TEM video for grain boundary sliding and crack propagations.
